# The lack of keratinized mucosa as a risk factor for peri-implantitis: a systematic review and meta-analysis

**DOI:** 10.1038/s41598-023-30890-8

**Published:** 2023-03-07

**Authors:** Basel Mahardawi, Sirimanas Jiaranuchart, Napat Damrongsirirat, Sirida Arunjaroensuk, Nikos Mattheos, Anupap Somboonsavatdee, Atiphan Pimkhaokham

**Affiliations:** 1 Department of Oral and Maxillofacial Surgery, Faculty of Dentistry, Chulalongkorn University, 34 Henri Dunant Road, Wangmai, Patumwan, Bangkok, 10330 Thailand; 2 Department of Dental Medicine, Karolinska Institute, Stockholm, Sweden; 3 Department of Statistics, Chulalongkorn Business School, Chulalongkorn University, Bangkok, Thailand

**Keywords:** Risk factors, Dentistry, Periodontics

## Abstract

This study aimed to investigate the effect of the lack of keratinized mucosa on the risk of peri-implantitis, while also accounting for possible confounding factors. A literature search was conducted in PubMed and Scopus, including human studies that assessed the presence and width of keratinized mucosa in relation to the occurrence of peri-implantitis. Twenty-two articles were included, and 16 cross-sectional studies we meta-analyzed. The prevalence of peri-implantitis was 6.68–62.3% on patient-level and 4.5–58.1% on implant-level. The overall analysis indicated that the lack of keratinized mucosa was associated with a higher prevalence of peri-implantitis (OR = 2.78, 95% CI 2.07–3.74, *p* < 0.00001). Similar results were shown when subgroup analyses were performed, including studies with a similar case definition of peri-implantitis (Marginal Bone Loss, MBL ≥ 2 mm) (OR = 1.96, 95% CI 1.41–2.73, *p* < 0.0001), fixed prostheses only (OR = 2.82, 95% CI 1.85–4.28, *p* < 0.00001), patients under regular implant maintenance (OR = 2.08, 95% CI 1.41–3.08, *p* = 0.0002), and studies adjusting for other variables (OR = 3.68, 95% CI 2.32–5.82, *p* = 0.007). Thus, the lack of keratinized mucosa is a risk factor that increases the prevalence of peri-implantitis and should be accounted for when placing dental implants.

Dental implants are the preferred choice to replace missing teeth and restore the function and aesthetics of the edentulous site^1^. Although implants placed in the maxilla and mandible have demonstrated high survival rates throughout the years^2,3^, clinicians, as well as patients, should be aware of the possible technical and biological complications that may take place during the postoperative period. Among the most important complications to consider are peri-implant mucositis and peri-implantitis, which necessitate critical care^4^. Peri-implant mucositis is defined as an inflammation limited in the soft tissue surrounding dental implants, characterized by bleeding on gentle probing with no detectable bone loss. On the other hand, peri-implantitis is accompanied by progressive bone loss, beyond the initial bone remodeling stage that occurs following implant placement, along with signs of inflammation in the peri-implant mucosa (i.e., bleeding on probing, suppuration)^5,6^. Peri-implantitis, in particular, can impact the health of the peri-implant tissue irreversibly and compromise the long-term survival of dental implants, even when treated^7^. Consequently, prevention and early risk assessment remain the best strategy in the case of peri-implant inflammations.

Peri-implantitis is a poly-microbial anaerobic infection^8^. Overall, the main contributor to the onset of this event is the accumulation of plaque, owing to the vast number of bacteria it contains^9,10^. Formation of plaque will lead to peri-implant tissue inflammation, which results in peri-implant disease if progression was not reversed^11,12^. Several studies have been conducted, aiming to assess the prevalence of peri-implantitis and identify potential risk indicators that facilitate the onset of peri-implantitis. The results of such studies vary substantially, probably due to inconsistent case definitions, as well as the different inclusion/exclusion criteria^13–16^. Nevertheless, a number of risk indicators for peri-implantitis have been proposed, some of them have been confirmed in multiple studies^17,18^, while others are still controversial and without wider consensus or final conclusions reached^14,19^.

The width of keratinized mucosa (KM) in one of the anatomic features of the peri-implant tissue has been suggested as one of the potential risk factors for peri-implantitis^19,20^. Numerous investigations have been performed, aiming to find any correlation between the presence/absence of keratinized mucosa and its width, with the prevalence of peri-implantitis. The results of these reports in the literature differ greatly, with some indicating that the lack of keratinized mucosa is associated with a higher prevalence of peri-implantitis^13,21^, some showing no impact^22–24^, and others concluding that the presence of keratinized mucosa may lead to an increased occurrence of peri-implantitis^25,26^. Furthermore, previous systematic reviews of the impact of KM on implant health have combined data from many different clinical scenarios (e.g., fixed dental prostheses, overdentures) and from patients under different maintenance conditions, hence limiting the conclusions by possible confounding factors^27,28^.

According to the statement of the World Workshop in 2017, the evidence on the effect of keratinized mucosa width in relation to peri-implantitis is still insufficient^29^. In addition, although studies have shown that the presence of keratinized mucosa might be essential for maintaining the health of the peri-implant tissue^28^, a direct relation between the lack of keratinized mucosa and the prevalence of peri-implantitis is still inconclusive, due to the absence of meta-analyses relating specifically to this event^30,31^.

Thus, the goal of this systematic review and meta-analysis was to collectively analyze the available evidence, investigating whether the lack of keratinized mucosa around dental implants may lead to a higher risk of peri-implantitis. In addition, this investigation aimed to further analyze groups of studies with a similar case definition of peri-implantitis, restorative protocols, and maintenance conditions, aiming to further reduce any influence of potential confounding factors.

## Results

### Search outcomes

**Figure 1:**
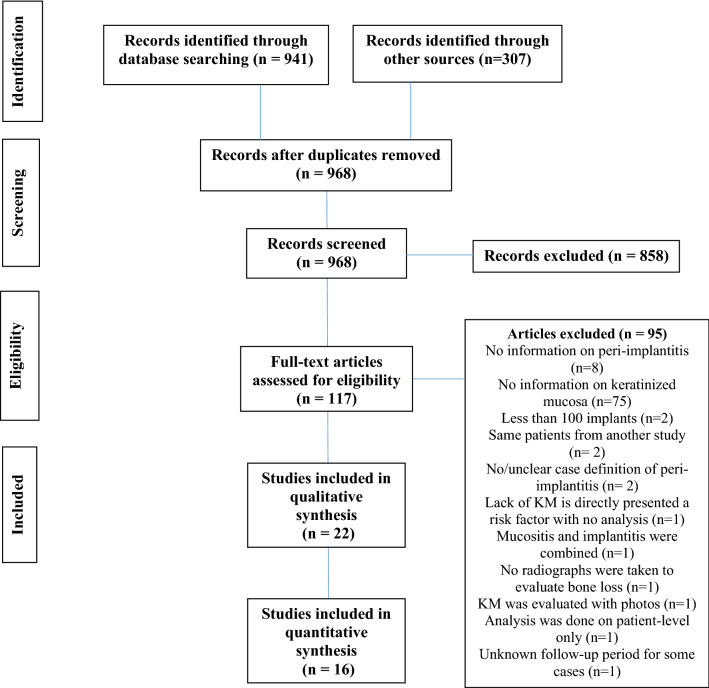
PRISMA flowchart of the search process and outcomes.

The search process resulted in identifying 1248 potential articles. After removing the duplicates and screening the titles and abstracts, one-hundred-and-seventeen studies reached the full-text assessment stage. Out of these, ninety-five articles did not meet the inclusion criteria (inter-examiner agreement ĸ = 0.80). As a result, twenty-two studies were included in this systematic review, and sixteen cross-sectional studies were meta-analyzed. Figure 1 is the PRISMA flowchart of the search process and results.

### Study characteristics

Included studies were published between 2006 and 2021. Two investigations were cohort^25,32^, and 20 studies were cross-sectional in their design. The total number of patients from the included papers was 4044 (21 studies), while the number of implants was 13,265. Eleven studies included fixed prostheses^13–15,19,20,22,26,33–36^, nine recruited patients with both fixed and removable prostheses^21,23,25,32,37–41^, one investigation included patients with implant-supported overdentures ^42^, while one study did not specify the type of restoration^43^. The follow-up period ranged between 1 year and up to more than 12 years^32^. All articles were in agreement regarding the presence of bleeding on probing (BoP) to define per-implantitis. However, the threshold of marginal bone loss (MBL) was inconsistent, revealing a wide range. Studies with more strict criteria defined peri-implantitis when MBL was > 0.5 mm^32^, while other investigations recorded this event when MBL was > 3 mm^13,19,20,25,34^. Schwarz defined peri-implantitis diagnosis when changes in bone level were seen, compared to the baselines^37^ (Supplementary Table 1). Based on different case definitions, the prevalence of peri-implantitis was 6.68–62.3% on patient-level, whereas it ranged between 4.5and 58.1% on implant-level. The keratinized mucosa (KM) threshold value varied between studies, with thirteen studies setting this value at 2 mm^13–15,19,20,22,26,33,34,40–43^, two at 1 mm^21,39^, while others defining this point as the presence/absence of keratinized mucosa^23,25,32,37,38^. One investigation reported the mean width of KM in healthy and peri-implantitis cases, demonstrating no significant difference between these values^36^. The odds ratio (OR) of peri-implantitis when there was a lack of keratinized mucosa width (KMW) ranged from 0.50 to 14.94^26,34^. Regarding cross-sectional studies, nine of them indicated that the lack of keratinized mucosa was not a significant risk indicator^14,22,23,33,36–38,42,43^, ten articles stated that the lack of KM is associated with a higher prevalence of peri-implantitis^13,15,19–21,34,35,39–41^, while one investigation pointed that the presence of keratinized mucosa is associated with an increased risk^26^. As for the cohort studies, one investigation indicated that the lack of KM is associated with a higher incidence of peri-implantitis, whereas one study showed that the presence of keratinized mucosa was associated with a significantly higher degree of bone loss^25^. Table 1. Summarizes the characteristics of the included studies.

**Table 1:** Characteristics of the included studies in this systematic review. KM: keratinized mucosa.

Study	Type	Number of patients/implants	Type of prosthesis	Average follow-up (year)	Prevalence of peri-implantitis (Patient-level) (%)	Prevalence of peri-implantitis (Implant-level) (%)	Keratinized mucosa cutoff point	Odds Ratio (implant- level) (95% CI)	Effect of KM on peri-implantitis
Alhakeem 2022	Cross-sectional	88/186	Fixed	7.3	–	9.6	< 2 mm or ≥ 2 mm	14.94 (4.12–54.14)	Lack of KM is associated with peri-implantitis
Vilarrasa 2021	Cross-sectional	169/311	Fixed	Minimum of 1 year	22.5	17.7	< 2 mm or ≥ 2 mm	4.85 (1.54–15.20)	Lack of KM is associated with peri-implantitis
Gharpure 2021	Cross-sectional	63/193	Fixed	1 year to > 10 years	–	19.17	< 2 mm or ≥ 2 mm	1.55 (0.73–3.27)	Lack of KM is associated with peri-implantitis
Gunpinar 2020	Cross-sectional	382/1415	Fixed/removable	3.79	36.9	21.7	< 2 mm or ≥ 2 mm	8.013 (3.494–18.380)	Lack of KM is associated with peri-implantitis
Wada 2019	Cross-sectional (retrospective)	543/1613	Fixed/removable	5.8	15.8	9.2	< 2 mm or ≥ 2 mm	2.32 (1.29–4.16)	Lack of KM is associated with peri-implantitis
Atieh 2019	Cross-sectional (retrospective)	188/423	Fixed	8.1	10.1	5.4	< 2 mm or ≥ 2 mm	6.29 (0.58–68.42)	Not significant
Romandini 2019	Cross-sectional	52/252	Overdenture on 4 implants	8.82	48.1	31.3	< 2 mm or ≥ 2 mm		Not significant
Kumar 2018	Cross-sectional	86/222	Fixed	Minimum of 5 years	62.3	58.1	< 2 mm or ≥ 2 mm	2.8 (0.6–17.5)	Not significant
Matarazzo 2018	Cross-sectional	211/748	Fixed	3.5	39.8	20.5	< 2 mm or ≥ 2 mm	1.658 (1.062–2.589)	Lack of KM is associated with peri-implantitis
Dalago 2017	Cross-sectional	183/916	Fixed	5.64	16.4	7.3	< 2 mm or ≥ 2 mm	1.69 (0.82–3.47)	Not significant
Ferreira 2015	Cross-sectional	192/725	–	4.02	8.33	9.39	< 2 mm or ≥ 2 mm	1.52 (0.86–2.70)	Not significant
Canullo 2015	Cross-sectional	534/1507	Fixed	Healthy: 5 Peri-implantitis: 5.9	10.3	7.3	< 2 mm or ≥ 2 mm	2.97 (1.67–5.31)	Lack of KM is associated with peri-implantitis
Vignoletti 2019	Cross-sectional	237/831	Fixed/removable	4.7	35	17.1	< 1 mm or ≥ 1 mm	2.48 (0.46–13.38)	Lack of KM is associated with peri-implantitis
Poli 2016	Cross-sectional	103/421	Fixed/removable		–	4.5	< 1 mm or ≥ 1 mm	3.99 (1.15–13.82)	Lack of KM is associated with peri-implantitis
Blume 2020	Cohort	145 implants	Fixed/removable	8.51 12.46	40.5	41.37	Presence/absence	4.489 (1.288–15.649)	Lack of KM is associated with peri-implantitis
Ahn 2019	Cross-sectional	111/209	Fixed/removable	Minimum of 7 years	–	16.7	Presence/absence	Presence of KM: 1.007 (0.384–2.638)	Not significant
Rokn 2017	Cross-sectional	134/478	Fixed	4.43	20.1	8.8	Presence (≥ 0.5 mm)/absence	3.89 (2.34–5.98)	Lack of KM is associated with peri-implantitis
Schwarz 2017	Cross-sectional	238/512	Fixed/removable	2.2	13.9	7.6	Presence/absence	1.74 (0.69–4.37)	Not significant
Koldsland 2011	Cross-sectional	109/354	Fixed/removable	8.4	20.4	11.4	Presence/absence	3.61 (1.31–9.92)	Not significant
Roos-Jansåker 2006	Cohort	218/982	Fixed/removable	9–14	6.68	6.72	Presence/absence	0.61 (0.36–1.03)	Not significant (Presence of KM was associated with more bone loss)
Canullo 2016	Cross-sectional	56/332	Fixed	Healthy 6.48 Peri-implantitis 5.94	–	37.6	Mean KMW healthy implants = 1.96 mm, Peri-implantitis = 1.96 mm	–	Not significant
Pimentel 2018	Cross-sectional	147/490	Fixed	300 implants < 5 years 190 implants > 5 years	19.1	9.2	≤ 2 mm or ≥ 3 mm	0.50 (0.27–0.92)	Presence of keratinized mucosa is associated with peri-implantitis

### Quality assessment

Supplementary Table 2 shows the quality assessment of the studies. Overall, the papers obtained scores between 6 and 8 points. Four articles scored 5/9 points^19,33,35,37^, which was mainly due to their case selection, in addition to the questionable assessment of the outcome.

### Results of the meta-analysis

Due to the insufficient number of longitudinal studies, only cross-sectional studies reporting on the prevalence of peri-implantitis and the impact of KM on this event were eligible for the meta-analysis.

#### Overall analysis

**Figure 2:**
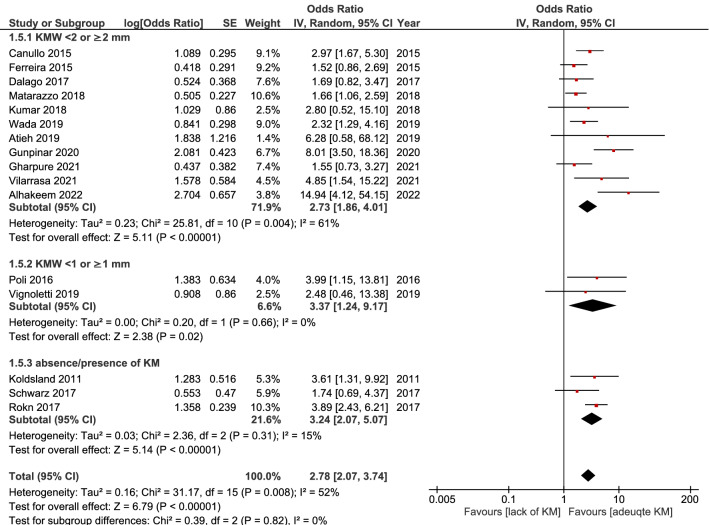
Forest plot to show the effect of lack of keratinized mucosa on developing peri-implantitis.

Sixteen studies were included in the overall meta-analysis^13–15,19–23,33–35,37,39–41,43^. A relatively high heterogeneity was found between studies (I^2^ = 52%), thus, the random-effect model was used. The results of this analysis indicated that the lack of keratinized mucosa was significantly associated with higher prevalence of peri-implantitis (OR = 2.78, 95% CI 2.07–3.74, *p* < 0.00001). A significantly increased risk of peri-implantitis was, likewise, noted when the KMW cutoff point was set at 2 mm (OR = 2.73, 95% CI 1.86–4.01, *p* < 0.00001), 1 mm (OR = 3.37, 95% CI 1.24–9.17, *p* = 0.02), as well as when KM was defined as present/absent (OR = 3.24, 95% CI 2.07–5.07, *p* = 0.008). (Fig. 2).

#### Similar case definition of Peri-implantitis

**Figure 3:**
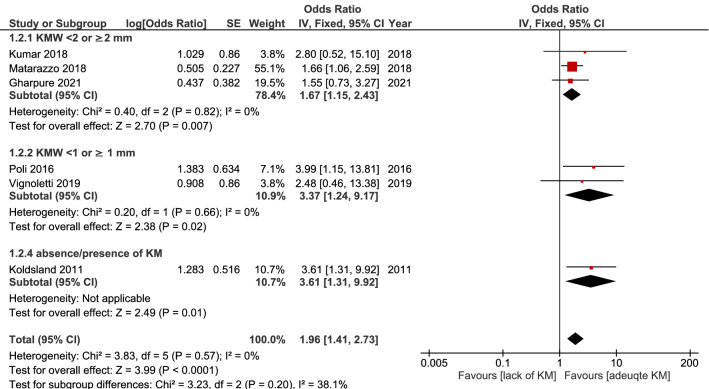
Result of the meta-analysis, including studies with similar case definition.

A meta-analysis was done on seven studies defining peri-implantitis when the degree of MBL was ≥ 2 mm^14,15,19,21,23,39^ (along with PPD and BoP). The fixed-effect model was implemented, as no heterogeneity was found (I^2^ = 0%). The results showed that the lack of KMW was associated with a significantly higher occurrence of peri-implantitis (OR = 1.96, 95% CI 1.41–2.73, *p* < 0.0001). (Fig. 3).

### Patients restored with fixed dental prosthesis

**Figure 4:**
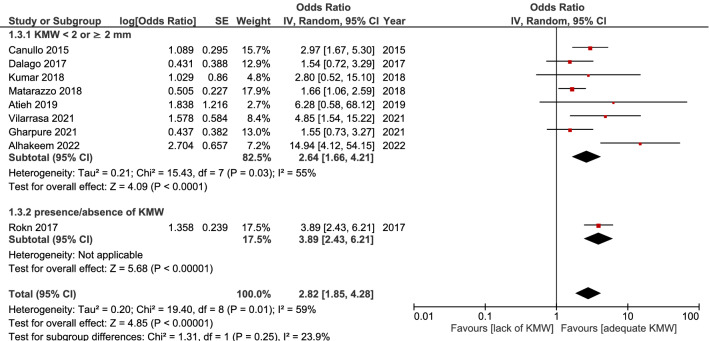
Result of the meta-analysis, including studies with fixed prosthesis only.

Investigations recruiting patients with fixed prostheses only were meta-analyzed^13–15,19,20,22,33–35^. Due to the notable heterogeneity among these studies (I^2^ = 59%), the random-effect model was applied. The lack of KM was again associated with a significantly increased prevalence of peri-implantitis (OR = 2.82, 95% CI 1.85–4.28, *p* < 0.00001). (Fig. 4).

### Patients under regular maintenance scheme

**Figure 5:**
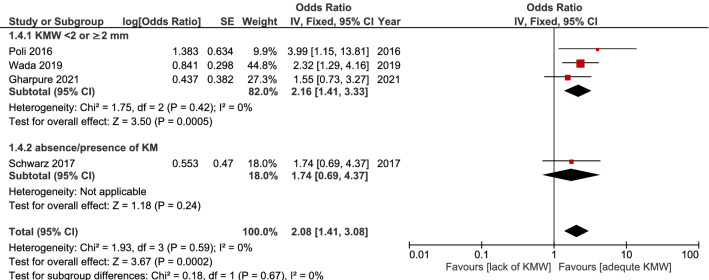
Result of the meta-analysis, including studies with patients under regular implant maintenance.

Four articles recruited only patients with regular recall maintenance appointments^19,21,37,41^. Meta-analysis of these studies revealed the lack of KM significantly raised the occurrence of peri-implantitis (OR = 2.08, 95% CI 1.41–3.08, *p* = 0.0002). No heterogeneity between studies was noted (I^2^ = 0%). (Fig. 5).

### Studies adjusting for other variables

**Figure 6:**
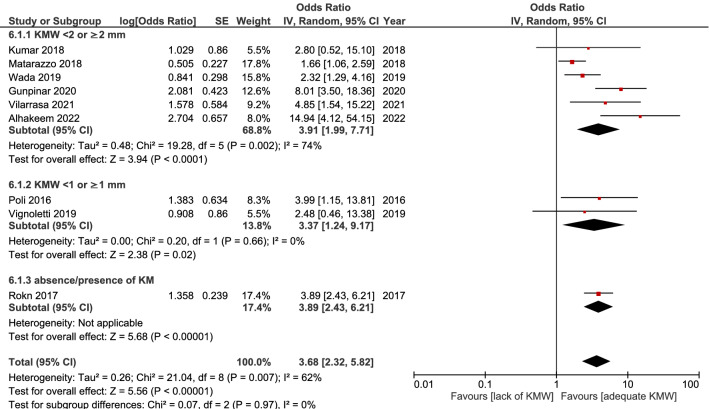
Result of the meta-analysis, including studies adjusting for other possible variables/factors.

Meta-analysis on studies accounting for other possible variables/risk factors (i.e., performing multilevel statistical analysis) was conducted. Nine studies were included^14,15,20,21,34,35,39–41^, with high heterogeneity among them (I^2^ = 62%). The results of this analysis also confirmed that an inadequate keratinized mucosa is associated with a significantly higher risk of peri-implantitis (OR = 3.68, 95% CI 2.32–5.82, *p* = 0.007). (Fig. 6).

### Publication bias

Although funnel plot of the overall meta-analysis showed a slight asymmetry in the distribution of studies with smaller effect (the bottom of the plot), sensitivity analysis by omitting each of the analyzed studies at a time did not cause a significant change in the results (OR = 2.53–2.96). Therefore, the reason for this could be the heterogeneity among the included studies^44^. Nevertheless, publication bias cannot be entirely ruled out (Supplementary Fig. 1).

### Level of evidence

Although the design of the included studies (cross-sectional) generally provides a low level of evidence, the results from all analyses were in agreement and showed consistency. In addition, the performed meta-analyses adjusted for several confounding factors. Therefore, based on the GRADE system, the certainty of evidence from the analyzed studies was judged to be “moderate”^45^.

## Discussion

The aim of this systematic review and meta-analysis was to provide a conclusive result on the effect of keratinized mucosa on raising the risk of peri-implantitis. Furthermore, by introducing a subgroup analysis where possible, this review attempted to limit the influence of possible confounding factors and expand the dimensions of the analysis to draw more reliable conclusions.

Peri‐implantitis is a pathological phenomenon taking place in the peri-implant tissue, manifested with inflammation of the mucosa around dental implants and a progressive loss of the supporting bone^29^. The prevalence of peri-implantitis differed largely among the included studies, ranging between 6.68–62.3% on patient-level and 4.5–58.1% on implant-level. As mentioned before, this is probably due to the numerous case definitions, setting different degrees of bone loss in each investigation, in addition to the divergent inclusion criteria, as well as a wide range of follow-up periods in these studies. This discrepancy in the prevalence of peri-implantitis was also presented in several systematic reviews^16,46,47^, confirming the previously stated reasons. Thus, future studies with more homogenous case definitions would offer a benefit in narrowing down this range and reaching more consensus between different populations.

Wennström and Derks concluded that the data on the significance of keratinized mucosa around implants are still scarce and no final conclusions could be drawn^27^. Similarly, a recent meta-analysis stated that the effect of a KM < 2 mm as a risk factor leading to peri-implant disease is still low^30^. On the other hand, another study pointed that the presence of KM < 2 mm showed a tendency towards having higher risk of peri-implantitis^31^. Other studies also stated the importance of keratinized mucosa for peri-implant tissue health and stability, showing more tissue inflammation when KM was insufficient^28,48^. The results of the current meta-analysis confirmed the effect of inadequate keratinized mucosa on the prevalence for peri-implantitis, and therefore, the presence of a sufficient amount of keratinized tissue should be taken into account when placing dental implants.

Based on the consensus report of the World Workshop in 2017, peri-implantitis is diagnosed when the degree of peri-implant bone loss is beyond the initial bone remodeling that occurs following implant placement^6^. Taking this into consideration, and knowing implant success has been earlier defined as having marginal bone loss of no more than approximately 1–2 mm during the first year^17,49^, further analysis was performed on studies defining peri-implantitis when MBL was ≥ 2 mm. This analysis also confirmed that insufficient KM was related to a higher prevalence of peri-implantitis. It is essential to note that when such analysis was carried out, no heterogeneity between studies was found (I^2^ = 0), which supports the previous assertion that a similar case definition of peri-implantitis would be of great value to reach more consistent results and have a better understanding of the nature of this pathology in future investigations.

Data used in the overall meta-analysis were from studies including patients with fixed, or both fixed and removable restorations. Conclusions on the effect of the type of prosthesis on peri-implantitis are still lacking, as several studies reported conflicting results^37,38,40,41^. Therefore, to further confirm the role of inadequate keratinized tissue on peri-implantitis, eliminating the possible effect of the type of prosthesis, additional analysis was conducted, including studies with a fixed type only. The result of this analysis also revealed that the lack of keratinized mucosa elevated the risk of peri-implantitis. One publication only was done recruiting patients with removable prosthesis^42^, indicating that the effect of KM was not significant. This was not in agreement with a previous report showing the effect of keratinized mucosa on the health of tissues around dental implants supporting overdentures^50^. Therefore, future studies considering the role of KM on peri-implantitis in patients with removable prosthesis are needed.

Regular periodontal maintenance has been stated as a crucial factor in preventing peri-implant disease^21,35^, since the main purpose of these appointments is to remove plaque from the periodontal tissue, which is a major contributor to peri-implant tissue inflammation^51–53^. Apart from professional plaque removal, regular maintenance visits raise patients' awareness towards the importance of cleaning the implant-surrounding tissues. However, the impact of keratinized mucosa on plaque accumulation and oral hygiene maintenance should also be taken into consideration, as it has been documented that a narrow KM (< 2 mm) leads to higher plaque accumulation and brushing discomfort^54^. Moreover, other investigations in relation to the involvement of keratinized mucosa as a factor affecting peri-implant health in patients with good oral care and under maintenance recalls were inconclusive^24,55^. Out of these points, a meta-analysis was conducted for studies explicitly reporting data from patients under regular maintenance programs. Ideally, one could analyze a cluster based on detailed plaque records, yet such data is scarcely reported in proper level of detail and a comparative manner, thus compliance with a maintenance program could be a surrogate as close as realistically possible at present. The outcome of this analysis further supported previous results, indicating that the lack of KM increased the prevalence of peri-implantitis, even for patients under regular maintenance. Thus, the lack of keratinized tissue should be considered a risk factor when placing dental implants, despite when a strict maintenance program is applied.

As several factors could be involved in developing peri-implants^14,41^, and since all studies focused on studying the influence of multiple elements, however, without accounting for other risk factors, a meta-analysis of studies considering the effect of other variables deemed necessary. The result of this analysis confirmed what was stated previously, showing a prevalence risk of peri-implantitis with an insufficient keratinized mucosa, consequently, supporting all the evidence provided earlier, based on other groups of studies.

The main strength of this investigation is the inclusion of a large number of studies. Moreover, conducting several meta-analyses of investigations with similar features increases the homogeneity and reduces the impact of potential confounding factors (i.e., case definition, type of prosthesis, maintenance frequency). This can increase confidence in the results and their relevance to clinical practice. Another positive point is that this study did not consider a certain keratinized mucosa cutoff point. All presented values were included and analyzed in subgroups, rather than focusing on a certain amount of KM, as this is still arbitrary, and an optimal width is yet to be determined. Thus, based on the relevant findings, it can be stated that the presence of a minimum amount of KM is essential for the health of peri-implant tissues. Nevertheless, certain limitations should also be thought of. More patient related and site-specific factors can be involved in the risk for peri-implantitis (e.g., implant location, oral hygiene, time-in-function, bone augmentation at the implant site), which this review could not account for, due to the inadequate data to conduct further analyses. Moreover, the majority of investigations were cross-sectional in their design. This allows only for assessing the prevalence of peri-implantitis and could lead to a certain degree of deviation in the outcomes based on the enrolled sample, which lowers the quality of evidence.

## Conclusions

Within the limitations of this study, it is indicated that the lack of keratinized mucosa is a risk factor that increases the prevalence of peri-implantitis. Clinicians should be aware of this factor when placing dental implants at a particular site. Future longitudinal studies are required, with homogenous case definitions and similar analysis, in order to confirm what is stated in the current meta-analysis and further identify more clinically relevant parameters and potential risk indicators.

## Methods

This study was registered in the PROSPERO database (CRD42022319868), and was conducted adhering to the Preferred Reporting Items for Systematic Reviews and Meta-analyses (PRISMA) guidelines^56^. The PICO protocol was followed to establish a suitable search question and include potential studies. The focused question of this systematic review was:

In partially or fully edentulous patients (P) receiving dental implants (I), does the lack of keratinized mucosa at the implant site increase the risk of peri-implantitis (O), compared to the presence of adequate keratinized mucosa (C)?

Population: partially or fully edentulous patients, in need for the replacement of their missing teeth and lack an adequate width of keratinized mucosa in their edentulous sites.

Intervention: the placement of dental implants to support fixed or removable prosthesis.

Comparator: partially- or fully-edentulous patients with adequate keratinized mucosa.

Outcome: occurrence of peri-implantitis.

### Search strategy

The literature search of this systematic review was carried out in PubMed (Medline) and Scopus databases. No limitations were applied in the search engine. The combination of free keywords and Medical Subject Heading search terms (i.e., MeSH) used in this study was (“peri-implantitis” OR “peri-implant disease” OR “peri-implant inflammation” OR “peri-implant pathology” AND “risk factors OR indicators” OR “dental implant prognosis”).

### Study selection

Included studies fulfilled the set criteria: human studies (cross-sectional, cohort, and case–control, whether prospective or retrospective), with at least 100 implants available for analysis^16,57^, and a postoperative follow-up of at least one year. Reports with no clear case definition or information related to peri-implantitis, or that did not investigate keratinized mucosa as a risk indicator for peri-implantitis were excluded from this systematic review.

Selection of potential articles was done independently by two reviewers (B.M and S.J), utilizing a website designated for screening systematic reviews (Rayyan, Qatar Computing Research Institute, Qatar Foundation)^58^. Any conflict that took place with regards to the inclusion/exclusion of some potential studies was solved by discussion or by consulting a third reviewer (A.P). The database search ended on November 13, 2022. A manual search in the references of included studies was done, attempting to find more articles that could be included.

### Data extraction

Data recorded from the included articles were study design, number of patients and implants, type of prosthesis (i.e., fixed, removable), mean or minimum follow-up time, case definition of peri-implantitis, prevalence of peri-implantitis on patient and implant levels, keratinized mucosa cutoff value (e.g., 1 mm, 2 mm, present/absent), odds ratio (OR) of peri-implantitis in relation to the effect of keratinized mucosa on this incidence, and the conclusion on the potential effect of keratinized mucosa from each study.

### Quality assessment

To evaluate the quality of the included cohort studies, the Newcastle Ottawa scale (NOS) was implemented^59^. The scale was modified for cross-sectional studies based on the design of this systematic review and following other published modifications^60,61^ (Supplementary File 1). NOS evaluates each study relying on 3 parameters: selection of the sample, comparability of the exposed/non-exposed groups, and assessment of the outcome of interest, giving each study a maximum score of 9 points. Studies scoring less than 6/9 points were considered to be of a low quality. The quality of each of the included studies was evaluated by the discussion between 2 reviewers (B.M, and S.J). A third reviewer’s opinion (N.M) was sought when a unanimous decision could not be made.

### Statistical analysis

Data on peri-implantitis, taking the keratinized mucosa into account, were recorded and analyzed. The odds ratio (OR) of peri-implantitis when the keratinized mucosa was below the set value in each paper, as well as the standard error (SE), were used to obtain the results of this meta-analysis. When a study reported the incidences of peri-implantitis to the total number of cases, the OR with its 95% confidence interval (CI) were calculated, and the SE was then calculated from the CI values^62^. The generic inverse variance method was applied by inserting the natural logarithm (ln) of the odds ratio, along with the related SE from the data of each study. The level of heterogeneity in the included studies was evaluated with the Chi^2^ test and I^2^ index. I^2^ values of 25%, 50%, 75% were considered as low, moderate, and high heterogeneity, respectively^63^. Whenever 50% or higher values were noted, the random-effect model was used, in order to reduce the bias resulting from methodological differences between studies. If no significant evidence of heterogeneity was found, the fixed-effect model was utilized. Subgroup and cluster analyses were performed to present the results from studies with similar keratinized mucosa cutoff value, similar definition of peri-implantitis with respect to the marginal bone loss, studies that included fixed prostheses only, as well as investigations that recruited only patients who were under a regular implant maintenance program. The outcomes of the meta-analyses were given as OR with its 95% CI. *P*-values of < 0.05 indicated statistical significance. Forest plots were created to illustrate the results of the meta-analysis for each group, and a funnel plot was generated to indicate whether potential study bias may exist. Moreover, sensitivity analysis was performed to confirm the robustness of this meta-analysis and to evaluate whether certain studies had any significant impact on the results. The data were analyzed using the Review Manager (RevMan) version 5.4 software (The Nordic Cochrane Centre, The Cochrane Collaboration, Copenhagen, 2020).

### Level of evidence

To assess the level of evidence from the included studies, the GRADE rating system was utilized^64^, which evaluates the quality of evidence as being “high”, “moderate”, “low”, or “very low” based on several factors (risk of bias, imprecision, inconsistency, indirectness, and publication bias). The certainty of evidence was judged based on the overall results of the analyzed subgroups and whether their outcomes are in line.

### Ethics approval and consent to participate

The article does not contain any experiments with human participants.

## Supplementary Information

Supplementary Information.

## Data Availability

The data that support the findings of this study are available from the corresponding author upon reasonable request.
