# Supplementary material for: The lack of keratinized mucosa as a risk factor for peri-implantitis: a systematic review and meta-analysis
Source: Sci Rep. 2023 Mar 7;13:3778. doi: 10.1038/s41598-023-30890-8 (PMC9992510; doi:10.1038/s41598-023-30890-8)
Supplement: Supplementary file 1 — Supplementary Information. [file 41598_2023_30890_MOESM1_ESM.pdf]

Study	Case definition
Alhakeem 2022	MBL ≥ 3mm, BoP/suppuration, PPD ≥ 4 mm
Vilarrasa 2021	BoP with/without PPD deepening, progressive MBL after 6 months of prosthetic loading.<br>Baseline radiographs not available: PPD >6 mm, MBL ≥ 3 mm from expected marginal bone remodeling
Gharpure 2021	BOP and/or suppuration, increase in PPD<br>signs of inflammation, MBL ≥2 mm compared with baseline radiographs.<br>If no baseline radiographs: MBL ≥3 mm, PPD 6 mm, and BOP
Gunpinar 2020	BoP and/or suppuration, PPD > 5 mm, MBL > 2mm
Wada 2019	BoP and/or Suppuration, MBL >1 mm
Atieh 2019	BoP and/or suppuration, MBL >2 mm
Romandini 2019	BoP and/or suppuration, PPD ≥ 4 mm, MBL ≥ 2 mm
Kumar 2018	PPD >4 mm, BOP/suppuration<br>MBL ≥2 mm compared to the time of functional loading.
Matarazzo 2018	Peri-implantitis: BoP/suppuration, MBL ≥2 mm.<br>Severe peri-implantitis: BoP/ suppuration, MBL ≥3 mm.
Dalago 2017	PD > 5 mm<br>BoP and/or suppuration, MBL > 2 mm
Ferreira 2015	MBL ≥ 2mm, PPD > 4 mm, BoP and/or suppuration.
Canullo 2015	MBL >3 mm, PPD ≥4 mm<br>BoP and/or suppuration
Vignoletti 2019	BOP and/or suppuration, MBL ≥ 2 mm
Poli 2016	peri-implant marginal bone loss ≥ 2.0mm<br>BoP and/or pus, PPD ≥4 mm
Blume 2020	BoP or exudation, MBL > 0.5 mm
Ahn 2019	BOP, PPD >5 mm, MBL >2 mm
Rokn 2017	BOP and/or suppuration.<br>MBL >2 mm
Schwarz 2017	BoP with/without pus.<br>Changes in bone level compared to the baseline.
Koldsland 2011	MBL ≥ 2.0 mm<br>BOP/suppuration,<br>PPD ≥ 4 mm.
Roos-Jansåker 2006	BOP and/or pus.<br>MBL ≥ 3 implant threads (≥ 3.1 mm)
Canullo 2016	PPD ≥ 4 mm, BoP, and/or suppuration,<br>MBL ≥3 mm compared with the radiograph taken at the time of the prosthetic replacement
Pimentel 2018	BoP and/or suppuration, PPD>4 mm, MBL ≥ 2 mm

Supplementary Table 1. case definition of peri-implantitis based on each study

Study	Selection				Comparability	Outcome			Total
	S1	S2	S3	S4	C	O1	O2	O3	
Alhakeem 2022	✸		✸	✸	✸✸	✸	✸	✸	8/9
Atieh 2019	✸	✸	✸	✸	✸✸		✸	✸	8/9
Vignoletti 2019	✸		✸	✸	✸✸	✸	✸	✸	8/9
Vilarrasa 2021	✸		✸	✸	✸✸	✸		✸	7/9
Wada 2019	✸		✸	✸	✸✸	✸	✸		7/9
Pimentel 2018	✸		✸		✸✸	✸	✸		7/9
Canullo 2016	✸		✸		✸✸	✸	✸	✸	7/9
Poli 2016	✸	✸	✸		✸✸	✸	✸		7/9
Canullo 2015	✸	✸	✸		✸✸	✸	✸		7/9
Ferreira 2015	✸	✸	✸		✸✸	✸	✸		7/9
Koldsland 2011	✸	✸	✸		✸✸	✸		✸	7/9
Gunpinar 2020	✸		✸		✸✸	✸	✸		6/9
Romandini 2019	✸		✸		✸✸	✸	✸		6/9
Ahn 2019	✸	✸	✸		✸✸		✸		6/9
Kumar 2018	✸		✸		✸✸	✸	✸		6/9
Matarazzo 2018	✸	✸	✸		✸✸	✸	✸		6/9
Gharpure 2021	✸		✸		✸✸	✸			5/9
Rokn 2017	✸		✸		✸✸		✸		5/9
Dalago 2017	✸		✸		✸✸		✸		5/9
Schwarz 2017	✸		✸		✸✸	✸			5/9
**Cohort studies**									
**Study**	**Selection**				**Comparability**	**Outcome**			**Total**
	**S1**	**S2**	**S3**	**S4**	**C**	**O1**	**O2**	**O3**	
Roos-Jansåker 2006	✸	✸	✸		✸✸		✸	✸	7/9
Blume 2020	✸	✸	✸		✸✸		✸		6/9

Supplementary Table 2. Quality assessment of the included studies

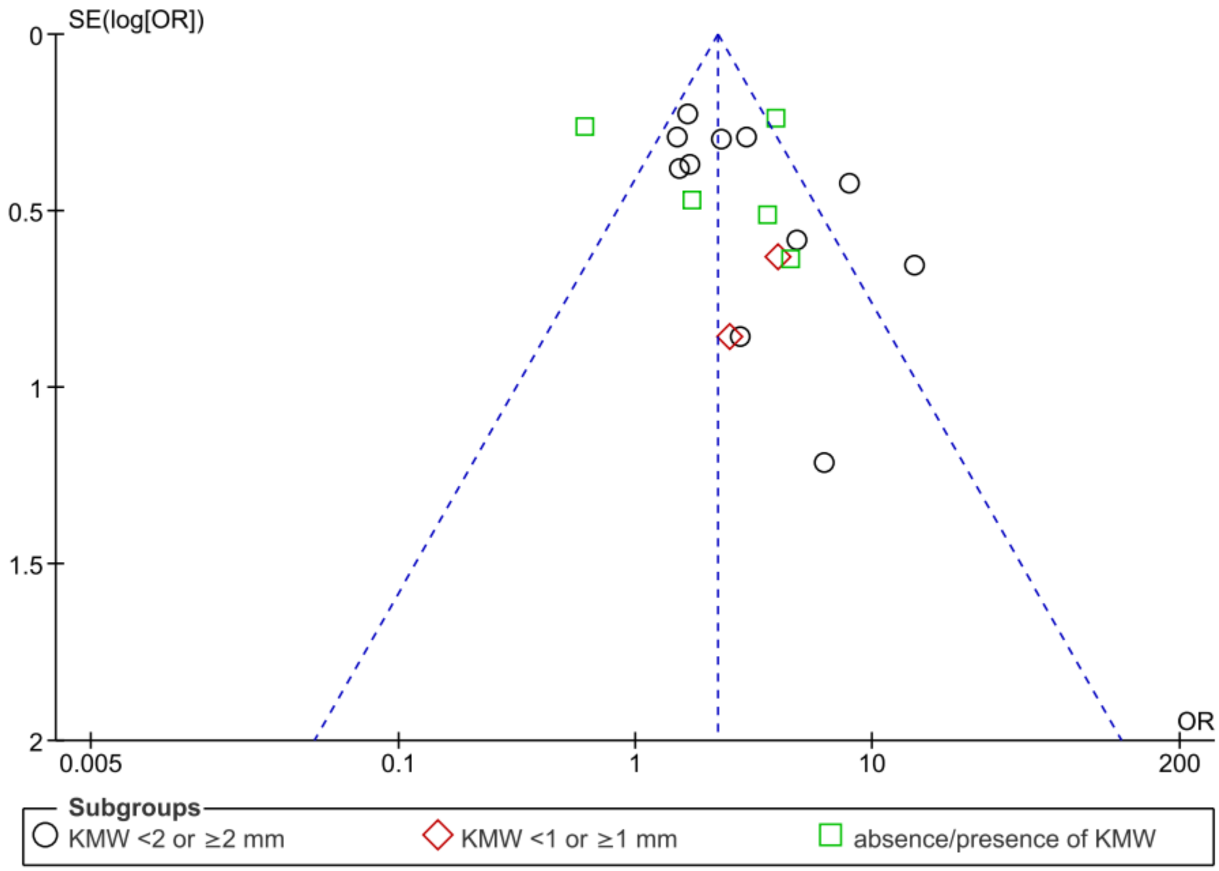

Supplementary Figure 1. Funnel plot to indicate potential publication bias in the included studies

NEWCASTLE – OTTAWA SCALE

(Modified for cross-sectional studies)

Selection (maximum 4 starts)

1. Adequate case definition with ascertainment of exposure
   a) yes, based on secure record (e.g., surgical records) ✸
   b) yes, based on structured interview/examination with validated measurement tool ✸
   c) written self-report
   d) no description
2. Representativeness of the selected cases
   a) truly representative of the average ________________ (describe) in the community ✸
   b) somewhat representative of the average _______________ in the community ✸
   c) selected group of users (e.g., nurses, volunteers)
   d) no description of the derivation of the cases
3. Selection of non-exposed/non-affected group
   a) drawn from the same community as the exposed group ✸
   b) drawn from a different source
   c) no description of the derivation of the non-exposed cohort
4. Definition of non-exposed (non-affected) group
   a) no history of disease (explicitly stated that non-exposed group have no history of this outcome) ✸
   b) no description of source

Comparability (maximum 2 starts)

1- Comparability of groups on the basis of the design or analysis
   a) Data/ results adjusted for relevant predictors/risk factors/confounders (e.g., age, sex, time since vaccination, etc.) ✸✸
   b) Data/results not adjusted for all relevant confounders/risk factors/information not provided.

Outcome (maximum 3 starts)

1- Ascertainment of outcome
   a) Secure record (e.g., surgical records) ✸
   b) Structured interview/examination where blind to affected/non-affected status ✸
   c) Interview/examination not blinded to affected/non-affected status, however, evaluated risk factors cannot be biased (e.g., age, sex, location, etc.) ✸
   d) Interview/examination not blinded to affected/non-affected status, however, examiner alignment and assessment (examiner calibration) was done prior to measurements. ✸
   e) Interview/examination not blinded to affected/non-affected status
   f) Self-report
   g) No description

2- Was follow-up long enough for outcomes to occur
   a) Yes (select an adequate follow up period for outcome of interest)✸
   b) No

3- Outcome/result is based on a justified sample (e.g., calculated sample size or suggested by official guidelines, such as consensus meeting)
   a) Yes ✸
   b) No

Supplementary File 1. Modified Newcastle-Ottawa scale for cross-sectional studies
